# Operationalising a social–ecological system perspective on the Arctic Ocean

**DOI:** 10.1007/s13280-017-0960-4

**Published:** 2017-10-26

**Authors:** Anne-Sophie Crépin, Åsa Gren, Gustav Engström, Daniel Ospina

**Affiliations:** The Beijer Institute of Ecological Economics, The Royal Swedish Academy of Sciences, Lilla Frescativägen 4, Box 50005, 104 05 Stockholm, Sweden

**Keywords:** Arctic Ocean, Integrated ecosystem-based management, Marine food web, Regime shifts, Social–ecological system

## Abstract

We propose a framework to support management that builds on a social–ecological system perspective on the Arctic Ocean. We illustrate the framework’s application for two policy-relevant scenarios of climate-driven change, picturing a shift in zooplankton composition and alternatively a crab invasion. We analyse archetypical system dynamics between the socio-economic, the natural, and the governance systems in these scenarios. Our holistic approach can help managers identify looming problems arising from complex system interactions and prioritise among problems and solutions, even when available data are limited.

**Electronic supplementary material:**

The online version of this article (doi:10.1007/s13280-017-0960-4) contains supplementary material, which is available to authorized users.

## Introduction

Global environmental changes challenge future human well-being (Steffen et al. 2015). Their impacts are substantial in the Arctic and likely to continue to affect Arctic development through changes in global climate and market prices of Arctic resources (e.g. Morgenroth 2014; Arctic Council 2016; Petrick et al. 2017; Troell et al. 2017). Climate change directly affects the Arctic through changed weather and sea ice conditions, impacting on water salinity, acidity, and temperature (Polyakov et al. 2012; Parkinson and Comiso 2013; Vihma et al. 2013). Sea ice reduction improves access to the Arctic, which favours shipping (IPCC 2014), tourism (Hamilton et al. 2005) and resource extraction (Petrick et al. 2017), despite substantial remaining challenges regarding profitability, safety and the lack of infrastructure (Petrick et al. 2017). The direct impacts on fish stocks will probably benefit fishing activities and aquaculture in the region (Troell et al. 2017). In contrast, indirect effects are less investigated. For example, climate change impacts elsewhere in the world are likely to affect Arctic economic activities through changes in the market prices of fish (Troell et al. 2017).

Attempts to manage the Arctic must account for the global context, multiple interactions between society and the environment, and possible unexpected abrupt changes (Arctic Council 2016). Lack of knowledge and data about these potential changes challenge management. We propose a framework to support management, which accounts for complex interactions between society and nature, possible abrupt change, and substantial uncertainties. Our approach merges Ecosystem-Based Management (EBM) with a Social–Ecological System (SES) approach. We call it *Integrated Ecosystem*-*Based Management* (IEBM).

EBM highlights key ecological interactions between biotic and abiotic components of ecosystems, and with society, rather than considering single issues, species or ecosystem services in isolation (McLeod et al. 2005; Arctic Council 2013). However, multiple and sometimes conflicting interests create the need for a SES approach to management, emphasising ecological interactions, socio-economic connections and a human-centred perspective. The SES approach recognises the essential role of ecosystems for life support and other services and the intricate relationships between ecosystems and society (Berkes and Folke 1998; Folke et al. 2011). While EBM has ecosystem management as main objective, *IEBM* focuses on sustainably managing the whole SES, ecosystems being an integrated part. The *IEBM* relies on an SES approach picturing one system where social and ecological patterns and processes interplay. However, *IEBM* takes into account the crucial role of ecosystems to provide goods, services and other relevant activities that contribute directly or indirectly to human well-being and Arctic sustainable development (MEA 2005). Hence, *IEBM* operationalizes an SES approach through a management lens.

Rational management decisions require knowledge about how the system will evolve, the impacts of human actions, the likelihood and impacts of different outcomes and their net benefits (Polasky et al. 2011a). Such knowledge is typically lacking, so decision makers must decide to either gather more information and delay action, or take actions based on poor information, both costly alternatives. Theories of resilience, decision making and complex systems, combined with the use of scenarios, like in *IEBM*, can increase awareness of the potential states, outcomes and the consequences of these outcomes under alternative decisions (Polasky et al. 2011a; Arctic Council 2016).

## Materials and methods

**Fig. 1:**
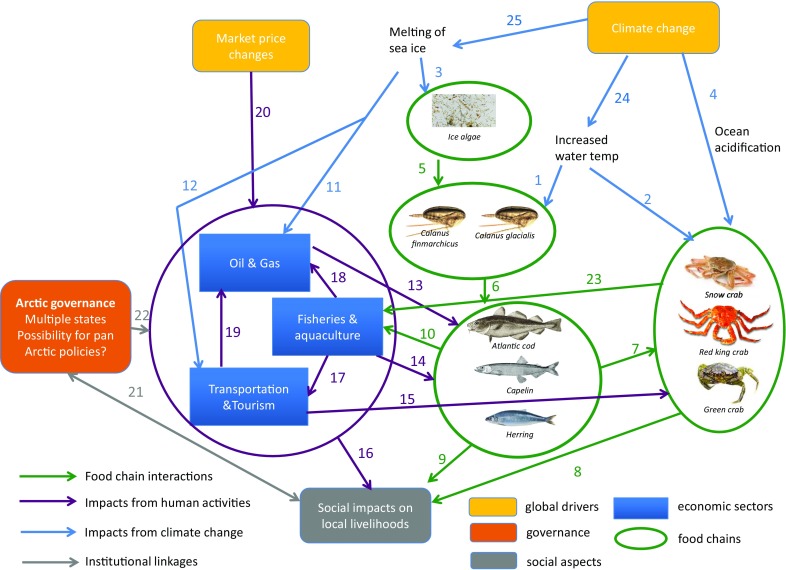
Social–ecological connections in the Arctic using the IEBM framework. The numbers refer to scientific articles supporting the arrows (Appendix S3)

**Fig. 2:**
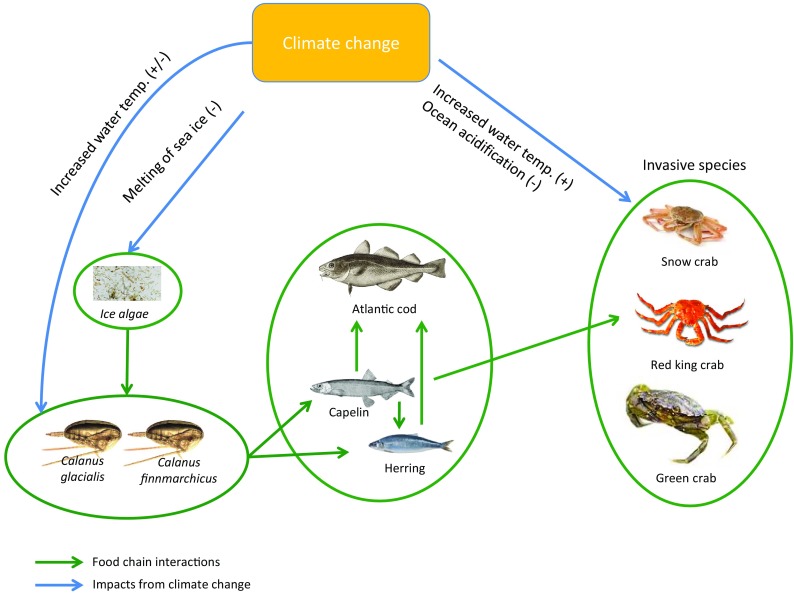
Food chain and climate change interactions in an Arctic marine ecosystem

We collected information about Arctic geophysical dynamics, food chains and socio-economic interactions through expert knowledge elicitation and literature studies (Appendices S1, S2 and S3). We synthesised this information (Table S1) to produce Figs. 1 and 2, representing interactions between socio-economic and natural elements of the Arctic seascape. To do so we used an SES approach (Berkes and Folke 1998) taking into account management objectives related to human well-being and ecosystems’ essential role for life support. Figure 1, and the information underlying it (e.g. models, empirical studies, narratives), forms what we call an *IEBM framework* for the Arctic Ocean. Figure 2 provides details on interactions between the climate and the Arctic food chain.

Based on insights from literature, expert knowledge and the *IEBM framework*, we identified six scenarios of plausible Arctic ecosystem change (Tables S2–S7). Scenarios are useful for analysing situations with large uncertainties (Carpenter et al. 2006). Recombining simultaneous trends, introducing surprise and low-probability high-impact events can help identify plausible but less expected futures (Postma and Liebl 2005). Our scenarios were simple fictive narratives of plausible Arctic ecosystem change that could occur within 50 years or earlier. We first identified the main drivers of change (such as climate change, management interventions, catastrophes, new policies), estimated potential impacts and pictured the system’s response. The Arctic marine species in their physical environment generate ecosystem services on which many economic activities rely, so we focused on the impacts of each scenario on ecosystem services and their response to climate change. Each scenario was then set in the context of the social–ecological interactions of the *IEBM framework* to identify key research questions (Appendix S4 and Tables S2–S7).

We selected two scenarios (Tables 1 and 2) featuring strong social–ecological interactions, complex dynamics, substantial uncertainties, potentially strong policy relevance and potential interactions between both scenarios through focus on a common key species. For both scenarios, based on all the background information gathered, we identified archetypes of large-scale social–ecological dynamics, which could lead to management failures (Homer-Dixon et al. 2015), inspired by the *Pathological Dynamics* framework (Peterson et al. 2017). By reducing the complexity of a rich body of systemic analyses of different global environmental problems down to a simpler common representation of interactions between the social, ecological and governance dimensions of a system, Peterson et al. (2017) isolate six stylised mechanisms that they call *pathological dynamics* (PDs). They draw on a diverse literature on global change, covering multiple disciplinary perspectives (economics, political science and ecology) to identify the specific governance and management challenges produced by these PDs, and strategies proposed to tackle them. Four PDs are applied to the scenarios in focus. *Long fuse big bang* occurs when slow gradual change leads to noticeable impacts only after a long time delay, making it difficult to address these impacts once they become apparent. *Rapid evolution* emerges when dynamic feedbacks within SES occur more rapidly than governance or management can respond. *Unforeseen processes* occur when important SES processes are unknown or novel. *Common dilemmas* arise when decisions by individual actors based on their personal costs and benefits lead to degradation of a shared resource because they do not account for the full impacts to the whole community (Peterson et al. 2017).

**Table 1:** Decrease in *Calanus glacialis* in favour of *Calanus finmarchicus.* Insights and key research questions derived from assessing scientific background information using an *IEBM* lens

Scenario 1. Decrease in *Calanus glacialis* in favour of *Calanus finmarchicus*	
Background information	References
The A grazer *Calanus glacialis* is an essential food source for many economically important fish species in the Arctic, including capelin, an important prey for Atlantic cod	Bogstad et al. (2000), Blachowiak-Samolyk et al. (2008), Søreide et al. (2008), and Eide (2017)
*Calanus finmarchicus* is an important food source for herring	Corten (2000)
Young herrings predate on capelin larvae	Huse and Toresen (2000)
*Calanus finmarchicus* is less lipid rich than *Calanus glacialis*	Søreide et al. (2008)
In a model simulation of climate change scenarios in the Barents Sea, the Atlantic zooplankton species *Calanus finmarchicus* increased approximately 20% and became more abundant in the east, while the Arctic zooplankton biomass (including *Calanus glacialis*) decreased 50%, causing the total simulated production to decrease	Ellingsen et al. (2008)
The red king crabs feed on capelin larvae	Mikkelsen (2013)
Oil spills risk harming vulnerable ecosystems in the Arctic Oil spills resulting from resource extraction and transportation seem to spread in different ways in open waters compared to different types of waters with sea ice	Nordam et al. (2017)

IEBM lens	
Insights	There will potentially be a reduction in the quantity and quality of zooplankton available for fish production in the Barents Sea
Key research questions	What are the implications of the reduction in quantity of zooplankton for fish production? What are the implications of the reduction in quality of zooplankton for fish production? What are the implications of a potential increase in herring predation on capelin larvae? What are the potential economic implications for the fisheries sector? What potential role can the red crabs play in changing the quantity of zooplankton? Does the zooplanktons vulnerability against pollution differ, and if so how? How does this affect local livelihoods, indigenous peoples and the local fisheries industries? Are there potential global repercussions?

**Table 2:** Increase in *red king crab* (*Paralithodes camtschaticus*). Insights and key research questions derived from assessing scientific background information using an IEBM lens

Scenario 2. Increase in red king crab (*Paralithodes camtschaticus)* biomass and distribution	
Background information	Reference
The red king crab benefits from increased water temperatures in the Arctic	Stoner et al. (2010)
The red king crab is an economically important species	Hjelstedt (2012)
Red king crabs predate capelin larvae	Mikkelsen (2013)
Capelin is a key food species for other economically important fish species, e.g. cod	Søreide et al. (2008)

IEBM lens	
Insights	An increase in the biomass of red king crabs, due to increased water temperature, can potentially reduce capelin production and thus also impact on the production of other fish species e.g. cod and herring
Key research questions	What are the implications of a potential increase and spread of red king crab in the Arctic for capelin production? What are the implications of a potential decrease in capelin production on the production of other economically important fish species like cod? What are the potential economic implications for the fisheries sector of cod and capelin? What are the implications for the co-management strategies of the cod and capelin fisheries? How does this affect local livelihoods, indigenous peoples and the local fisheries industries? Are those activities resilient to such change and could they seize the opportunity to produce King Crab instead? Are there potential global repercussions?

## Results

Figure 1 summarises the *IEBM framework* of interactions of human activities, ecosystem dynamics and governance with potential impacts of climate change and other global change. Figure 2 expands parts of Fig. 1 to highlight how climate change can impact the Arctic ocean food chain.

The dominant zooplankton *Calanus glacialis* is an essential food source for many Arctic marine species of fish (Blachowiak-Samolyk et al. 2008; Søreide et al. 2008). Meanwhile, a warmer climate will likely favour the less lipid-rich grazer *C. finmarchicus* (Ellingsen et al. 2008), which could have negative impacts on higher trophic levels (Falk-Petersen et al. 2007, 2009; Steen et al. 2007). In particular it could negatively affect Capelin (*Mallotus villosus*), Herring (*Clupea harengus*) and Atlantic cod (*Gadus morhua*) (Bogstad et al. 2000).

Table 1 illustrates the scenario “Decrease in *Calanus glacialis* in favour of *Calanus finmarchicus*”. It focuses on how climate change could trigger a regime shift in the zooplankton species composition from *Calanus glacialis* to *Calanus finmarchicus* with potential spin-off effects on the Arctic marine food web. In this scenario, the biomass of *Calanus finmarchicus* increases by approximately 20% and becomes more abundant in the East, while the Arctic zooplankton biomass decreases by 50%, causing the total production to decrease (Ellingsen et al. 2008).

Given the importance of *Calanus glacialis* for feeding capelin (Orlova et al. 2010), a decrease in the quantity of *Calanus glacialis* could negatively impact the capelin stocks, potentially leading to fewer fish and maybe lower quality of fish, due to different fat contents in both *Calanus* species (see e.g. Scott et al. 2000; Falk-Petersen et al. 2009). These changes could have a knock-on effect on cod and herring because cod prey on capelin (Søreide et al. 2008), and herring feed on *Calanus finmarchicus* (Corten 2000) and capelin larvae (Huse and Toresen 2000). Furthermore, inflow of warm water to the Barents Sea (Toresen and Østvedt 2000; Sætre et al. 2002) favours herring. These changes would have repercussions on the markets for these two important commercial fish species (FAO 2017).

Economic activities will also most likely increase the spread of non-native marine organisms (Lewis et al. 2003) and invasive species like crabs in the Arctic (Ruiz and Hewitt 2009). Two recently introduced crab species could become economically important but also disrupt the balance in the Arctic marine food chains: the Red king crab (*Paralithodes camtschaticus)* (Hjelstedt 2012) and the snow crabs (*Chionoecetes opilio)* (Alvsvåg et al. 2009). However, increasing ocean acidification, resulting from elevated CO_2_ in the atmosphere, could inhibit growth of shells leading to crab mortality (Doney et al. 2009; Long et al. 2013).

Table 2 illustrates the scenario “Increase in red king crab”, picturing the potential effects of an increase in the invasive species red king crab (*Paralithodes camtschaticus)*. In this scenario, this introduced species has grown to be of great economic importance in parts of the Arctic. The population of red king crab supports a valuable fishery in the Barents Sea, (ex-vessel value of 26 million USD in 2011, Hjelstedt 2012). However, the benthic communities in northern Norway and the Kola Peninsula in Russia are facing significant disturbance from the red king crab, including the destruction of benthic communities (Jørgensen and Primicerio 2007) and predation on capelin eggs (Mikkelsen 2013).

**Fig. 3:**
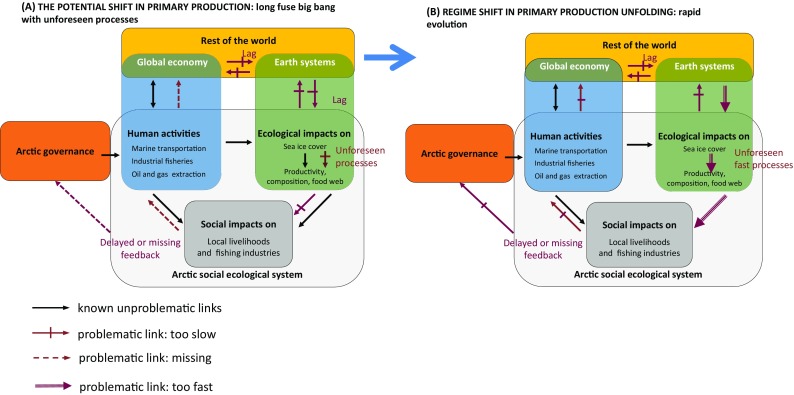
Pathological dynamics in a potential regime shift in primary production

Figure 3 illustrates *PDs* unfolding in scenario 1 during A) the build-up process before a regime shift—*long fuse big bang*—and B) the regime shift—*rapid social*-*ecological evolution*. Panel A refers to processes of slow gradual change with impacts only noticeable after a long time delay. Time lags characterise the accumulation of greenhouse gases, with impacts manifesting now in the melting of the Arctic summer ice sheet and through changes in temperature, salinity and light penetration. Panel B captures the possible rapid shift in zooplankton composition that might ensue once ice sheet conditions change enough to critically affect the timing of life cycles between ice algae and *Calanus glacialis* (Søreide et al. 2010). The dynamic *unforeseen processes* (not illustrated here) could also be an important feature inducing feedback between scenarios 1 and 2, via further unanticipated ecological changes throughout the food web, and the effects of invasive species.

**Fig. 4:**
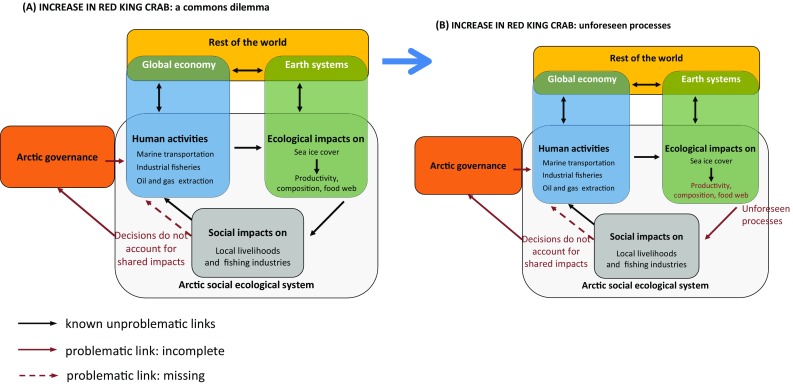
Pathological dynamics in an increase of red king crabs

Figure 4 illustrates two *PDs* characterising scenario 2. (A) a *common dilemma* occurs when individual actors introduce the red king crab for their own personal winning without accounting for its impacts on the ecosystem or other actors. The existing institutions do not properly regulate these kinds of novel activities and the species is introduced. During phase (B) the species has become so abundant that it could generate *unforeseen processes* in Arctic food webs due to surprising interactions with other species.

## Discussion

### Scenario analysis using the framework

Despite the uncertainty about the likelihood of the scenarios studied and how they may combine, they reveal only three types of impacts on economically important fisheries: (1) an increase in targeted fish stocks, (2) an increase in crab stocks associated with a decrease in fish stocks, and (3) a decrease in crab and fish stocks. In the scenarios where most wild species would decrease, aquaculture may become an alternative for livelihood and food production. However, food originating from substantially decreasing fish stocks may become more expensive and growing conditions for the cultivated species in a new environment with warmer and potentially more acidic water may not be optimal (Troell et al. 2017).

Examining jointly both scenarios of focus reveals that competition from red king crabs with associated increased predation pressure on capelin larvae could further reinforce a decreasing trend in fish stocks from a shift in *Calanus* species (Mikkelsen 2013). Such predation occurs already and is likely to increase unless negative impacts on crab survival, for example from ocean acidification, outweigh the advantage of increased water temperature for crab expansion. Simultaneously, activities like transportation, tourism and resource extraction will generate more pollution in Arctic waters (Nordam et al. 2017) and atmosphere (Law et al. 2017). Differences in physiology (Scott et al. 2000) between *Calanus* species may generate different responses to the expected increased water pollution, an aspect that Ellingsen et al. (2008) do not account for in their simulations.

The economic implications for fishermen would depend on the size of the decrease in fish stock, whether invasive species like crabs could be profitably fished, whether change in zooplankton also affect these species, and whether alternative activities are available to fishermen, in case fishing becomes unprofitable. The overall effect on fish stocks and fisheries depends on the size of these changes in relation to large natural variations in the Arctic seascape. Fisheries are exposed to substantial price variations that may impact the sector more than the large, but expected, variations in the ecosystem (Eide 2008, 2017; Troell et al. 2017).

Most impact assessment studies (e.g. ACIA 2004) showing increases in fish stocks account only for the *direct* impacts from global warming on fisheries, through changes in water temperatures and other oceanographic changes. Analysing the *indirect* impacts, using the *IEBM framework,* reveals that climate change could put synchronised pressure on different parts of the system, which all separately lead to potential downward trends in fish stocks. Hence, extremely low fish stocks relative to natural variation could become a more frequent reality if the extremes of all these different trends happen to align.

Moreover, spin-offs from climate change impacts in other parts of the world will likely affect economic and governance systems in the Arctic. For example decreasing fish stocks in the rest of the world combined with collapse of agricultural activities in some regions (IPCC 2014) is likely to increase demand for Arctic fish stocks, leading to price increase and thus incentives to fish more.

Having identified these important links qualitatively, through expert knowledge elicitation, the next steps would be to quantify the impacts of each scenario on all parts of the system. The information provided in our framework could facilitate such an exercise by highlighting non-obvious dynamic social, economic and ecological links that need to be included.

A thorough quantification exercise requires substantial baseline data and data on how much changes in stocks of zooplankton, pollutants, invasive species, among others, are likely to alter the system. This is costly and time consuming. The *IEBM framework* helps in separating destabilising dynamics (positive feedbacks) from dynamics that stabilise the system (negative feedbacks), thus identifying potentially risky situations, like when multiple, seemingly independent, processes reinforce each other and risk to move the system towards a new trajectory. For example, a decrease in *Calanus*, due to pollution, could coincide with substantial crab invasion, while ocean acidification remains at a manageable level for the crabs, potentially leading to fish stock collapses. The framework could also provide guidance on how to prioritise which additional information should be collected, in particular, which variables to monitor regularly.

### Pathological dynamics in climate change scenarios

Examination of Fig. 1 in the *IEBM* framework reveals that the *long fuse big bang* dynamics, leading to a shift in primary production studied in scenario 1, could change food chain interactions in the Arctic marine food webs (Corten 2000; Søreide et al. 2008), triggering another *PD* called *unforeseen processes* (Peterson et al. 2017). Changes in food webs and ecosystem composition, in response to geophysical changes associated with climate change, are uncertain, but could have substantial impacts on the stocks of commercial fish species. The difficulty of anticipating such changes makes an appropriate social response challenging.

In scenario 1, dynamics during the regime shift may become too rapid to control through governance or management, creating a process of *rapid social*–*ecological evolution* (Fig. 3b; Peterson et al. 2017). The main difficulty is to address the speed of social–ecological innovation, and improve the rate and ability of social–ecological system to adapt to rapid change. For example, if *Calanus finmarchicus* becomes rapidly the dominant zooplankton in the Arctic, and this change triggers a substantial drop in the main commercial fish stocks, the economic activities linked to these fish stocks and the institutions that regulate them may not be able to react quickly enough to the ecological change, leading to inappropriate management.

All three *PD*s mentioned exhibit missing or delayed feedbacks between social impacts and governance. They all possess problematic links between human actions, ecological impacts and social impacts because these are either lagged, too rapid or unexpected.

In contrast, scenario 2 reveals *common dilemma* dynamic, which happens when the incentives shaping agents’ behaviours do not align with the collective consequences of their actions. Key features are substantial mobility or spatial diffusion of the resource and expectations about other people’s behaviour. This scenario could also trigger *unforeseen processes*.

Peterson et al. (2017) identified two potential solutions common to *long fuse big bang*, *unforeseen processes* and *rapid evolution*. These are *monitoring* and *reduced pressure on the environment*. *Monitoring* is also a potential solution to *common dilemmas*. The Arctic Council has emphasised the need to better monitor the Arctic through its working group “Arctic Monitoring and Assessment Programme”. *Monitoring* by collecting information about complex ecosystem dynamics would improve understanding of the problematic links present in all four *PD*s. Such understanding could reveal early warning signs of impending change, and provide incentives for more prosocial behaviour. The *IEBM* could help prioritise among possible monitoring activities.

In contrast, *reduced pressure on the environment* is a precautionary approach to management that would decrease the risk of operating in poorly understood, underexplored environments. Precaution has been previously advocated when managing systems with endogenous risks of abrupt changes affecting system dynamics (Polasky et al. 2011b).

*Scientific research* and *strengthening institutional linkages* are common solutions to *rapid evolution* and *unforeseen processes*. *Scientific research* would help understand the complex food chain processes involved in a shift between *Calanus* species and thus maybe identify potential interventions, while *strengthening institutional linkages* would improve the likelihood to be able to observe and address unusual change in species interactions in a coherent way. *Strengthening institutional linkages* could also help address *common dilemmas*.

## Concluding remarks

The *IEBM framework* developed here and tested using scenario analysis and the *PD* framework, helps pinpoint management challenges from a shift in *Calanus* species and increase in red king crabs, prioritise among these challenges and identify appropriate responses when information is scarce. The *IEBM framework* cannot currently support precise quantified predictions of future outcomes; such predictions would remain speculative due to all the unknown and unknowable variables in the system. However, the way we link together Arctic system elements using the *IEBM framework* and both scenarios studied stem from solid scientific grounds.

The *IEBM framework* improves system understanding and helps identify novel links and drivers of change, revealing potentially non-obvious problems that could occur for example when changes are multiple and synchronise, so-called *multiple whammies* (Homer-Dixon et al. 2015). Arctic marine ecosystems and local fishers are accustomed to rapid and substantial change and show good resilience to direct impacts of Arctic climate change. In contrast, indirect impacts like changes in food chains or invasion of new species could alter the picture radically. The Arctic social–ecological system may cope with one disturbance at a time, but capacity to cope with simultaneous disturbances may be limited. Reinforcing feedback loops could trigger rapid change, for example if invasive species, pollution and ocean acidification all simultaneously contribute to decreasing fish stocks.

The *IEBM framework*, together with the *PD* framework, reveal synergetic solutions to multiple problems. Together these frameworks help shrink the range of reasonable interventions when information is scarce by focusing on the dynamics that could lead to major system destabilisation, rather than trying to marginally improve production output. When information is scarce and costly, it may be difficult to build a detailed simulation model to provide quantified predicted impacts of change. Potential solutions can emerge, even with limited information, because the frameworks focus attention on important connections, differentiate stabilising and destabilising elements in the system and provide information on potential areas for intervention. The *IEBM framework* can support stakeholders and managers to identify priority areas for information collection, prioritise among problems, identify synergetic solutions to multiple problems and select appropriate policy responses. For example, it may be relatively easy to reach an agreement to keep local pollution at low levels through regulations and choose location of transport routes and resource extraction strategically, so that they do not coincide with the most sensitive sites for fish stock reproduction. In any case, it is essential that local fishers and the regional fisheries industry develop resilient strategies to deal with potential impacts of climate change. Adopting a system view and using tools like the *IEBM framework* could help identify threats and opportunities and steer actions to improve the likelihood of desirable outcomes.

Compared to other management tools currently available, the *framework for IEBM* provides a more holistic approach and set of solutions. In contrast, EBM does not incorporate dynamics in the socio-economic part of the system, computable general equilibrium models exclude most natural dynamics and marine spatial planning discards non-geographical interactions. The IEBM can address all these different aspects and can also incorporate all these different tools, while still being able to provide decision support even with limited information.

Future extensions of the framework could include nested models, to use within the framework (e.g. models developed in Eide 2017, Petrick et al. 2017) and a user-friendly interface to test strategies and their performance against particular objectives. Such interface could include an Arctic marine spatial planning tool (Edwards and Evans 2017) and available datasets (Godøy and Saadatnejad 2017).

Other scenarios like sea-level rise, coastal erosion or reduced or increased oil extraction could have important impacts for some communities already at a short time scale. While the current study does not address such changes, the IEBM could also accommodate those, along with more detailed studies of the social and economic drivers of human behaviour that impact on the system’s trajectory. A starting point could be to add details from other models developed in this special issue (Eide 2017).

## Electronic supplementary material

Below is the link to the electronic supplementary material.

Supplementary material 1 (PDF 1076 kb)
