# Supplementary material for: Operationalising a social–ecological system perspective on the Arctic Ocean
Source: Ambio. 2017 Oct 26;46(Suppl 3):475–85. doi: 10.1007/s13280-017-0960-4 (PMC5673876; doi:10.1007/s13280-017-0960-4)
Supplement: Supplementary file 1 — Supplementary material 1 (PDF 1076 kb) [file 13280_2017_960_MOESM1_ESM.pdf]

Ambio
Electronic Supplementary Material

This supplementary material has not been peer reviewed.

Title: **Operationalising a social-ecological systems perspective on the Arctic Ocean**

Authors: Anne-Sophie Crépin, Åsa Gren, Gustav Engström, and Daniel Ospina

Appendix S1: Comprehensive overview of the Arctic social-ecological system from literature studies

Arctic ecosystems and climate change

The lipid-rich Arctic grazer *Calanus glacialis* forms up to 80% of zooplankton biomass in the Arctic shelf seas and feeds on ice algae, the key primary producers. *Calanus glacialis* is an essential food source for many Arctic marine species, including fish (Blachowiak-Samolyk et al. 2008; Søreide et al. 2008). Currently, melting ice floating in from other areas affect water temperature. When climate change stops this process, the less lipid-rich grazer *C. finmarchicus* is likely to replace *C. glacialis* (Ellingsen et al. 2008), which would have direct negative impacts on higher trophic levels (Falk-Petersen et al. 2007; Steen et al. 2007; Falk-Petersen et al. 2009).

Herrings (*Clupea harengus*) are essential prey fish for higher trophic levels. They predate on zooplankton and are thus potentially sensitive to changes in *Calanus* species. Inflow of warm water to the Barents Sea favours the climate dependent herring recruitment (Toresen and Østvedt 2000; Sætre et al. 2002).

Capelin (*Mallotus villosus*) is an important forage fish, and Atlantic cod (*Gadus morhua*) its main predator (Bogstad et al. 2000). Capelin distribution and migration depend on ocean currents and water masses, making the species potentially sensitive to the impacts of climate change on these parameters. Recruitment and growth of Atlantic cod are positively correlated with sea temperature (Ottersen et al. 1998) so global warming is likely to affect the Barents Sea cod fisheries through water temperatures and other oceanographic changes. Although increased fluctuations in stock biomass and stock age composition are predicted,

these will probably remain limited compared with normal environmental fluctuations in this area, (Eide 2017; Troell et al. 2017). However, indirect effects like changes in prey, competition, and parasites are less investigated. For example food competition and herring predation on capelin larvae may generate collapses in the capelin stock, and other factors seem to influence capelin population dynamics too (Gjøsæter et al. 2015).

Ware et al. (2014) predicted that by the end of the century, maximum sea surface temperatures in e.g. Svalbard could rise to 12.5°C. Several marine invasive species, like crabs, could expand to sub-Arctic and Arctic waters even under moderate climate change scenarios (De Rivera et al. 2007). The European green crab (*Carcinus maenas*) can tolerate a wide range of salinities and survive in temperatures of 0 to 30 °C (Cohen et al. 1995). This 'global invader' has had ecological and economic consequences in other regions and could have in the Arctic too (Hines et al. 2004). The Red king crab (*Paralithodes camtschaticus),* introduced in the Barents Sea over 40 years ago, benefits from warmer ocean temperatures and it has a currently estimated population above 12 million in the Barents Sea alone (WWF Norway). The Red king crab supports valuable fisheries (Hjelstedt 2012). However it may also pose a risk for native species due to predation and habitat destruction (Falk-Petersen et al 2011, Jørgensen 2005; Jørgensen & Primicerio, 2007, Mikkelsen 2013). The first *S*now crabs (*Chionoecetes opilio)*, recorded in 1996 in the eastern part of the Barents Sea, are now a major part of this ecosystem, but our knowledge of this new inhabitant is still poor (although see Hjeltsen 2014). The snow crab has a high commercial potential, which Norway and Russia have started to explore (Pettersen 2014). However, it is unclear just how beneficiary different crab populations will be to society until the impacts of the increasing crab invasion on other parts of the Arctic ecosystem can be more thoroughly assessed. Furthermore, ocean acidification, resulting from elevated CO_2 in the atmosphere, has increased by about 30 % since the beginning of the Industrial

Revolution (Guinotte and Fabry 2008) and could inhibit growth of shells leading to crab mortality (Doney et al. 2009; Long et al. 2013).

Economic activities in the Arctic seascape and climate change impacts

Despite the lack of strong pan-Arctic fisheries, some areas – like the Barents Sea – enjoy a significant level of fishing activities. In addition to direct probably positive impacts of climate change on fish stocks (Eide 2017; Troell et al. 2017), substantial change could occur in primary (phytoplankton) and secondary (zooplankton) production (see e.g. Falk-Petersen et al. 2007). Indirect impacts of climate change on fisheries through markets are also expected (Troell et al 2017).

Arctic aquaculture is a prominent and growing industry representing about 2% of worldwide volume, which is comparable to the total production within the European Union. Salmon farming in Norway makes up the bulk of the production while there is a minor aquaculture production in Iceland and Russia. Climate change impacts on water temperature, salinity, oxygen concentration and quality will likely affect aquaculture organisms' growth and health (Troell et al. 2017).

Current shipping activity in the central Arctic Ocean is low but expected to increase to and from Arctic harbours and eventually between continents due to sea ice reduction (Christensen et al. 2014; IPCC 2014; Petersen 2014; Troell et al. 2017). Climate change will open up new shipping routes and increase the use of current routes for transit navigation. A redistribution of tourists from warm areas to colder destinations is also likely (Hamilton et al. 2005). Ultimately the patterns of tourism depend on demand, supply, services, and available infrastructure. Substantial challenges remain regarding safety, and economic profitability of transports and tourism activities, due to extreme weather, drifting icebergs, ice thickness, and changes in travelling time.

Current physical conditions and profitability limit resource extraction in the Arctic. Climate change will positively affect these conditions and extend the areas in which extraction is

technically feasible and economically profitable thanks to sea-ice decreases and cheaper transports (Morgenroth 2014; Petrick et al. 2017). However resource extractions will remain challenging and risky activities (Petrick et al 2017).

Arctic shipping for transportation, tourism, fisheries, and resource extraction increases black carbon emissions and fossil fuels use, creating a reinforcing feedback loop to the climate (Dalsøren et al. 2013) and could have a significant impact on the atmospheric composition in the northern polar region (Law et al. 2017). Current emissions are connected to fisheries (50%, Corbett et al 2010) and to some extent tourism (Ødemark et al 2012). Oil spills resulting from resource extraction and transportation seem to spread in different ways in open waters compared to waters with sea ice (Nordam et al. 2017). Spills could harm vulnerable ecosystems and spawning grounds for important fish stocks (e.g. Vesterålen/Lofoten), essential for fisheries and tourism (Noring et al. 2016).

Marine transportation and resource extraction also increase noise pollution, which is likely to affect the Arctic marine ecosystem, marine mammals in particular (ACCESS 2014a;b; Vedenev et al. 2014). In addition, the external hull and ballast tanks of vessels operating in ice-covered waters can support many non-native marine organisms (Lewis et al. 2003), and spread more invasive species in the Arctic (Ruiz and Hewitt 2009).

Different hierarchical levels of regulation operate in the Arctic, ranging from regional to international. They result in multilateral and national agreements, with hard and soft laws, guidelines and recommendations. Transboundary stock distributions, and changing management practices present ongoing challenges for governance for example.

Gaps in governance for the Arctic include the lack of unified observation system, regional fisheries management systems, and basic infrastructures, gaps in the IMO polar code regarding climate change effects, a fragmented approach to regulations for resource extraction, and poor escape, evacuation and rescue facilities. (ACCESS 2014c).

Appendix S2: Expert knowledge elicitation: key socio-ecological links in changing Arctic and emerging risks

We performed expert knowledge elicitation among a transdisciplinary group of researchers collaborating within the European Union Seventh Framework project Arctic Climate Change Economy and Society (ACCESS, 2011-2015, Dnr:265863) within the call "The Ocean of Tomorrow". We distributed a questionnaire (See S2A) by email to the whole consortium (65 researchers from 27 partners in 9 countries) and collected answers by e-mail and during the second ACCESS general assembly held in Villanova i la Geltru, Spain in March 2013. The researchers in the group came from diverse disciplinary backgrounds including oceanography, economics, social anthropology, political sciences, ecology, and more. The group also contained some stakeholders. Some of the respondents had coordinating responsibilities either for particular research tasks of the project, whole work packages or even the whole consortium. The respondents were asked individually to identify what activities within their own area of expertise (climate change, marine fisheries, oil and gas extraction, shipping, and governance) could affect the other areas, and how. Similarly, they were also asked to identify how developments on other areas could affect their area. In total we received 30 responses including responses from all work-package leaders and other key participants.

The questionnaire information was complemented with results from several group exercises with ACCESS researchers during ACCESS general assemblies in March 2013 (Cambridge, UK) and 2014 (Vilanova i la Geltru, Spain), as well as two synthesis meetings in September 2013 (Bremen, Germany) and 2014 (Stockholm, Sweden). During these sessions participants had the opportunity to identify and discuss possible cross sectoral interactions between ACCESS sectors of activities (See Appendix S2B). They were asked to suggest potential interactions, which were synthesized in a big chart of interactions (Table S1). We also benefitted from the exercises performed by students at two graduate courses organized by the ACCESS consortium (Bremen, September 2013, ACCESS 2013; and Stockholm September 2014, Ospina and Crépin 2014).

In addition we performed a traditional literature study, using a variety of sources including, ACCESS project results disseminated through Newsletters, scientific deliverables, publications and synthesis to identify essential variables and build a picture of their possible interactions. (S1)

A) Questionnaire given at the ACCESS general assembly in Barcelona, March 2013

This questionnaire was distributed by email to all researchers participating in ACCESS ahead of the second ACCESS general assembly in Barcelona March 2013. The answers were collected by e-mail and on paper at the general assembly itself and respondents were given incentives to respond in the form of a surprise (some chocolate bars) handed out at the general assembly.

The respondents were asked to answer three questions in relation to their ongoing activities within the ACCESS project. In total 65 questionaires were given to the participants of which 30 were returned. This is a relatively low response rate. However, among the respondents we obtained responses from all key sources, such as work package leaders and other project participants with some responsibilities to overlook the work performed in the project. This increases our confidence that we managed to collect most of the relevant knowledge within the project consortium at that time.

Questionnaire

Purpose: Identifying links/flows/connections between the different sectors included in ACCESS, to contribute to the construction of a social-ecological systems framework. A deliverable to be provided by WP5

Question 1:
Which sectors within ACCESS (fisheries, oil and gas, climate, transport and tourism, governance) are you familiar with?

Question 2:
*Which activities in **your** sector(s) affect **other** sectors, and how (please give references if you have any)?*

Question 3:
*Which activities in **other** sectors affect **your** sector(s), and how (please give references if you have any)?*

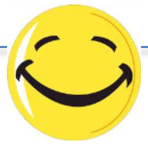

Thank you!

Turn please

Please fill in your name and e-mail address, in case we need to contact you for further clarifications and/or questions

Name: ____

E-mail address: _____________________________________

The information will be treated anonymously and the only persons having access to names will be Anne-Sophie Crépin, Åsa Gren and Gustav Engström. If you have any questions, please feel free to contact us at:

asa.gren@beijer.kva.se

asc@beijer.kva.se

gustav.engstrom@beijer.kva.se

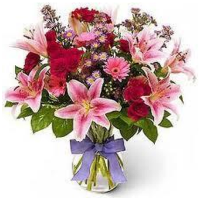

Thank you again for your contribution!

Anne-Sophie Crépin, Åsa Gren and Gustav Engström

B) Instructions to break out groups at the ACCESS general assembly, Barcelona March 2013.

We organised group sessions at the ACCESS general assemblies in Barcelona (March 2013, See instructions below) and Cambridge (March 2014) and organised two workshops in Bremen (September 2013) and Stockholm (September 2014). During these sessions participants had the opportunity to identify and discuss several possible interactions between ACCESS sectors of activities so called cross-sectoral interactions. They were asked to suggest potential interactions on post it papers that they could stick on a big chart of interactions (See Table S1). We also benefitted from the exercises performed by students at two graduate courses organized by the ACCESS consortium (Bremen, september 2013, D6.251 and Stockholm September 2014, D6.253)

Instructions for break out group 1:

Establishment of infrastructure in the Arctic Ocean (e.g. Oil platforms, aquaculture)

Your group should discuss cross-sectoral questions related to the establishment of infrastructures in the Arctic Ocean, in particular oil platforms, aquacultures and other infrastructures related to resource extraction. You should discuss aspects concerning impacts from and to the environment as well as social, political and economic aspects. To guide the discussion you could address some of the sub questions below or discuss other aspects that the group finds relevant to the topic.

The group leader is responsible for moving the discussion forward, in addition we suggest that you take 2 minutes to identify the following roles in the group:

- a note-keeper who documents the discussion
- a time keeper who manages time to make sure you can to address all the relevant aspects
- a rapporteur who will shortly present the results of your discussion at the plenary session tomorrow morning.

To help you address as much as possible during the short time period that you have we suggest that you use some of the following techniques, which are not exclusive:

- You can split into smaller groups for part of the time to address different questions
- You can collect people's ideas on stickers that you together organize and group
- You can do short roundtables so that everybody is given a chance to shortly express their view on the topic
- Etc.

What guidelines could ACCESS produce regarding establishment and management of infrastructures in the Arctic Ocean?

Reflections on the following issues (suggestions for smaller break out groups maybe) may help come up with such guidelines:

a. What is the environmental impact of such infrastructures? How is it affected by the particular characteristics of the Arctic environment? Which of those characteristics are the most relevant?
b. What are the potential impacts of these infrastructures on other economic activities? (Are there particularly important regions for these activities? What are the needs for infrastructure on land? What are the profit margins of such activities in this environment?)
c. Are the current rules and regulations regarding establishment, management and contingency planning sufficient?
d. Will climate change trigger the need for changes in existing rules and institutional settings? Is there need for contingency planning in case of accidents? In which regions will new opportunities occur due to climate change?

Instructions for break out group 2:

Arctic Marine transportation

Your group should discuss cross-sectoral questions related to Arctic marine transportation including for example goods transportation through the Arctic, fishing boats going for harvest, transportation of oil and minerals from the Arctic, tourism cruising. You should discuss aspects concerning impacts from and to the environment as well as social, political and economic aspects. To guide the discussion you could address some of the sub questions below or discuss other aspects that the group finds relevant to the topic.

The group leader is responsible for moving the discussion forward, in addition we suggest that you take 2 minutes to identify the following roles in the group:

- a note-keeper who documents the discussion
- a time keeper who manages time to make sure you can to address all the relevant aspects
- a rapporteur who will shortly present the results of your discussion at the plenary session tomorrow morning.

To help you address as much as possible during the short time period that you have we suggest that you use some of the following techniques, which are not exclusive:

- You can split into smaller groups for part of the time to address different questions
- You can collect people’s ideas on sheets of paper that you together organize and group
- You can do short roundtables so that everybody is given a chance to shortly express their view on the topic
- Etc.

What guidelines could ACCESS produce regarding marine transportation in the Arctic Ocean?

Reflections on the following issues (suggestions for smaller break out groups maybe) may help come up with such guidelines:

e. What is the environmental impact of marine transportation? How is it affected by the particular characteristics of the Arctic environment? Which of those characteristics are the most relevant?
f. What are the potential impacts on other economic activities? Will there be competition or synergies between transports trough the Arctic and transports of Arctic goods to outside markets?
g. Are the current rules and regulations regarding establishment, management and contingency planning sufficient?
h. Will climate change trigger the need for changes in existing rules and institutional settings? Is there need for contingency planning in case of accidents? In which regions will new opportunities occur due to climate change?

Instructions for break out group 3:

Sustainable use of resources and services from Arctic ecosystems

Your group should discuss cross-sectoral questions related to sustainable use of resources and services from Arctic ecosystems including fisheries and tourism. You should discuss aspects concerning impacts from and to the environment as well as social, political and economic aspects. To guide the discussion you could address some of the sub questions below or discuss other aspects that the group finds relevant to the topic.

The group leader is responsible for moving the discussion forward, in addition we suggest that you take 2 minutes to identify the following roles in the group:

- a note-keeper who documents the discussion
- a time keeper who manages time to make sure you can to address all the relevant aspects
- a rapporteur who will shortly present the results of your discussion at the plenary session tomorrow morning.

To help you address as much as possible during the short time period that you have we suggest that you use some of the following techniques, which are not exclusive:

- You can split into smaller groups for part of the time to address different questions
- You can collect people's ideas on stickers that you together organize and group
- You can do short roundtables so that everybody is given a chance to shortly express their view on the topic
- Etc.

How can we continue to use and benefit from Arctic marine resources and ecosystem services in a sustainable way? Can ACCESS produce guidelines?

i. Are there institutional challenges for sustainable use? (e.g. collective access to the resource which is hard to restrict)
j. What are the economic challenges? (profit margins, quotas constraints, fuel cost, salaries, etc.)
k. How is climate change expected to impact on these activities?
    i. Direct impacts (weather changes, ice melting, etc.)
    ii. Indirect ecosystem impacts (changes in habitats, species migrations, regime shifts (tipping points)
    iii. Indirect economic impact (increased demand for fish from the "last" productive stock, substantial global population increase, tec.
l. Will these changes trigger the need for changes in existing rules and institutional settings?

Appendix S3: References to Figure 1

1. Ellingsen et al. 2008, Falk-Petersen 2007.

2. Cohen et al. 1995 (green crab), De Rivera et al. 2007 (several species).

3. Söreide et al. 2010.

4. Long et al. 2013; Doney et al. 2009 ; Steinacher et al. 2009.

5. Söreide et al. 2010.

6. Blachowiak-Samolyk et al. 2008; Søreide et al. 2008; Falk-Petersen et al. 2009; Scott et al. 2000; Falk-Petersen et al. 2007; Steen et al. 2007.

7. Mikkelsen 2013.

8. Pettersen 2014 (snow crab); Hjelstedt 2012 (red king crab).

9. Stammler-Gossmann 2014; Troell et al 2017.

10. Troell et al 2017.

11. Petrick et al. 2017.

12. Hamilton et al. 2005a and 2005b.

13. Noring et al. 2016; Nordam et al. 2017. Wilkinson et al. 2017.

14. Troell et al. 2017.

15. Lewis et al. 2003; Lewis et al. 2004.

16. ACCESS 2015a. Wilkinson et al. 2017.

17. Table S1.

18. Noring et al. 2016; Olsen et al 2007.

19. Table S1.

20. Morgenroth 2014; Petrick et al. 2017; Troell et al. 2017.

21. Table S1.

22. ACCESS 2012; ACCESS 2015b.

23. Hines et al. 2004; Hjelstedt 2012; Mikkelsen 2013; Pettersen 2014

24. Hoegh-Guldberg, and Bruno 2010.

25. Polyakov et al. 2012; Stroeve et al 2012a, 2012b; Wadhams 2012; Wadhams et al. 2011; Wang and Overland 2012.

Appendix S4: Creating and analysing scenarios

To illustrate the usefulness of the IEBM framework presented in Figures 1 and 2 of the paper, we identified six potential scenarios of change based on the insights obtained through expert-knowledge elicitation (S1 and S2). The scenario are presented in Tables S2-S7. Each scenario presents a fictive short narrative of plausible Arctic ecosystem change that could occur in a relatively near future as a consequence of climate change, for example "decrease in *Calanus glacialis* in favour of *Calanus finmarchicus*". The narrative is supported by background information with references to the scientific literature that supports the most important elements of the particular scenario.

These scenarios were then placed back in the context of the broader socio-ecological interactions of the IEBM framework (Figure 1), in order to identify key research questions for each scenario. The scenario "decrease in *Calanus glacialis* in favour of *Calanus finmarchicus*" presented in table S2 illustrates the potential effects of a regime shift, when changing the composition of key zooplankton species, switching from *Calanus glacialis* to *Calanus finmarchicus.* The implications of this ecological shift were further detailed by considering insights from a fisheries model focusing on the relation between *Calanus* spp and the economically important Atlantic cod, under climate change (Eide 2017).

The background information forms the essential assumptions for scenarios of change in the Arctic. To develop scenarios it is crucial to find out how the basic assumptions of each scenario will impact on essential ecosystem services in order to find out the full range of impacts of potential changes. First the scenarios should identify the main drivers of change (e.g. climate change, management intervention, catastrophe, new policy instrument), what potential impacts may arise from these drivers, and the system's response to these drivers. For example the species of the Arctic Ocean, interacting with each other and the physical environment generate the ecosystem services on which many economic activities rely. Hence it is of interest to assess the impact of climate change on them in order to identify potential future change in ecosystem service generation in the Arctic Ocean. Using a

systems perspective and an ecosystem service lens to assess potential impacts of climate change helps identify different potential scenarios of change and identify knowledge gaps that need to be addressed to aim for a sustainable management of Arctic fisheries.

We identified six hypothetical scenarios of possible change in the Arctic marine ecosystem driven by climate change. Two of these scenarios focus on changes in zooplankton production and four scenarios on changes in presence of Arctic species like crabs.

At least two major potential impacts of climate change on the Arctic zooplankton community are worthy of further investigation in the context of sustainable cod and capelin management: First is the potential mismatch between the two peaks on primary production by ice algae, and the reproductive cycle of key Arctic grazers such as the *C. glacialis* (Søreide et al. 2010), resulting from the reduction in sea ice thickness and coverage area. The second one is the potential switch from *C. glacialis* to the less lipid-rich Atlantic grazer *C. finnmarchicus*, due to competitive advantages of the latter species under climate change. According to a model simulation of climate change scenarios (Ellingsen et al. 2008), Atlantic zooplankton species increased approximately 20% and became more abundant in the east, while the Arctic zooplankton biomass decreased 50%, causing the total simulated production to decrease. Herring, another economically important species, is an effective converter of zooplankton into fish, and is thus also potentially sensitive to change in zooplankton production. Furthermore, herring is favoured by inflow of warm water to the Barents Sea (Stephens and Krebs 1986; Sætersdal and Loeng 1987; Hamre 1994; Toresen and Østvedt 2000; Sætre et al. 2002), and since young herrings predate on capelin larvae, a potentially significant effect on these populations associated to climate change might take place. Given the importance of *Calanus* for feeding capelin and herring, a decrease in the quantity of feed is likely to negatively impact these stocks, potentially leading to fewer fish and maybe also lower quality of fish, due to the difference in fat content between the two *calanus* species (see e.g. Falk-Petersen et al. 2009; Scott et al. 2000).Tables S2 and S4 illustrate the rationale behind two possible scenarios of zooplankton change. An integrated ecosystem-based management perspective

on these scenarios helps provide the “key research questions” associated with each scenario and provides substantial support to also answer those questions in a way that incorporate relevant but non obvious aspects. For example would fish feeding to a larger extent on *Calanus finnmarchicus* respond in a different way to potential oil spills compared to fish feeding mostly on *Calanus glacialis*?

Several crab species have or are becoming dominant species in the Arctic marine ecosystem. The European green crab will potentially benefit from increased shipping and transportation in the Arctic. The green crab has also been estimated to have the potential to expand to sub-Arctic and Arctic waters even under moderate climate change scenarios (De Rivera et al. 2007). Also, it has been shown that conditions under which species can reproduce are more relevant in estimating establishment potential than physiological tolerances. Based on this assumption Ware et al. (2014) predicted that by the end of the century, maximum sea surface temperatures in areas like Svalbard are predicted to rise beyond 10°C (12.5°C), thus rendering a number of non-indigenous species, including the European green crab, able to reproduce there. What are the potential implications for Arctic ecosystems of crossing that temperature threshold, in combination with increased shipping? Table S5 illustrates a possible scenario of change related to the European green crab.

The red king crab is another introduced species, which has grown to be of great economic importance in parts of the Arctic. The population of red king crab supports a valuable fishery in the Barents Sea, representing an ex-vessel value of 150 million NOK in 2011 (Hjelstedt 2012). However, it has also been confirmed that the benthic communities in northern Norway and the Kola Peninsula in Russia are facing significant disturbance from the red king crab (Joergensen and Primicerio 2007). In order to estimate the total economic impact of the red king crab on the Arctic social-ecological system, both the pros and the cons of the crab on the Arctic ecosystem must be assessed. This, apart from the profits of catching and selling the crabs, also entails assessing the connection between the destruction of benthic communities by the red king crab and the production of other economically important

species, such as the capelin, since concern has been expressed about the predation on capelin eggs by the red king crab (Mikkelsen 2013). Table S3 illustrates a scenario where the red king crab increases substantially, Table S5 emphasizes an increase of the European green crab while Table S6 focuses on snow crabs.

Ocean acidification is another process that only recently has been shown to have potentially great impact on a multitude of marine species. The oceans have turned 30% more acidic since the beginning of the Industrial Revolution (NOAA 2010). Besides increased acidity of the ocean, this also entails other changes in the sea's chemistry, such as robbing the water of important minerals that marine creatures need to grow, especially those with shells. Long et al. (2013) determined the effects of long-term exposure to near-future levels of ocean acidification on the growth, condition, calcification, and survival of juvenile red king crabs (*Paralithodes camtschaticus*), and Tanner crabs (snow crabs, *Chionoecetes bairdi*) and found that both species survival decreased with pH, with 100% mortality of red king crabs occurring after 95 days in pH 7.5 water. More research is needed to add the potential effects of ocean acidification to the already complex context of climate change in the Arctic marine ecosystems, especially in the context of crustaceans. However Table S7 makes an attempt to identify a scenario of increased ocean acidification.

Table S1: Interactions between sectors of activities in the Arctic seascape as assessed during ACCESS expert elicitation process: how elements in columns affect elements in row

	Geophysical environment	Marine ecosystem	Fisheries	HC extraction	Shipping and tourism	Local people	Governance	Rest of the world
**Geophysical environment**		Change (+/-?) in water salinity affect primary production and fishes | Increased ocean acidification impact on crustaceans | Reduced sea-ice extent and duration, affects phyto and zooplankton cycles	Lower ice extent increase potential zones of fisheries	Lower ice extent increase possibilities for energy extraction | Increased icing as closed ice cover / ice recedes (?)	Lower ice thickness increase transportation & tourism / longer seasons of navigation | Artic persived pristiness attracts tourism	Change in cultural meaning for indigenous peoples	Climate change impacts increase pressure on more unified governance for the Arctic	Ice melting leads to sea level rise | Impacts for climate change in the Arctic incentivize regulation in rest of the world
**Marine ecosystem**			Fish stocks depend on marine primary productions		Artic perceived pristiness attracts tourism		Transboundary nature of marine ecosystems requires international governance (given condition?)	Impacts for climate change in the Arctic incentivize regulation in rest of the world
**Fisheries**						Fisheries are an important source of income for local people	Negative changes in fisheries put pressure on governance	Important for global food security
**HC extraction**		HC extraction increases pressure (risks?) on marine ecosystem | Oil and wind platforms provide hard structure facilitating (?) invasive species movement, negatively affecting the ecosystem	Competition or cooperation for transport routes and infrastructures?		Competition or cooperation for transport routes and infrastructures? | Increase marine transportation due to HC extraction, damages tourism due to increased risks	Important source of income for local people (?) | Negative effects (risks?) on local livelihoods and economies	Probably oil/gas still source of conflicts outside EEZs?	(Still too expensive to have an impact on global price) | New source of HC, enables continued CO2 emissions**
**Shipping and tourism**	Increased transportation leads to more black carbon depositation	Invasive species increase with more maritime transportation | Transportation increases pressure (risks?) on marine ecosystem | Tourism increase pressure on fish stocks	Competition or cooperation for transport routes and infrastructures?	Competition or cooperation for transport routes and infrastructures? | Synergies in infrastructure development (also: SAR)**	Increase marine transportation due to trade routes, damages tourism due to increased risks	Source of income and activities? | Negative impacts on local/indigenous communities (?) | SAR / Telecom (?)	Increased traffic demands regulations and management (?)	Increased transportation increases CO2 emissions** (Or shorter routes reduce CO2 emissions?)
**Local people**		Local people also use the marine ecosystems (?)					How can minorities influence Arctic governance? | Inclusion of 'traditional knowledge' in policy making (?)	
**Governance**	Monitoring for appropriate implementations and Indicators	Ecosystem management and tools tailored for the Arctic | Monitoring for appropriate implementations and Indicators	Regional management system | Monitoring for appropriate implementations and Indicators	Polar code | Standarising national regulations on hydrocarbons | Port of refuge to minimize environmental damage from potential off-shore spill | Monitoring for appropriate implementations and Indicators	Polar code | Monitoring for appropriate implementations and Indicators	Indigenous people affected by different levels of governance (?) | How to compensate subsistance harvesters without conventional monetary methods? | How to document cultural resources to protect them from damage or loot, without taking away its secrecy?		Potential considerations of fisheries regulations elsewhere in the world?
**Rest of the world**	Global climate change is driver of changes in Arctic geophysical environment	Global sources of pollution affect Arctic marine ecosystems?	Global demand for fish drives fisheries companies into the Arctic	Global demand for HC drives extractive companies into the Arctic	Global interest in new shipping routes and Aarctic tourism	Global diffusion of values and ideas incluences local people	International conflicts over different topics can affect negotiations on the Arctic	

** **Potential reinforcing feedback**

positive effect
negative effect
mixed or unclear effects
needs clarification
posed as question
governance need
governance already developed

Table S2: Scenario 1, decrease in Calanus glacialis in favour of Calanus finnmarchicus

<table>
<tr><th colspan="2">Scenario 1. Decrease in Calanus glacialis in favor of Calanus finnmarchicus</th></tr>
<tr><td>Background information</td><td>Reference</td></tr>
<tr><td>The arctic grazer Calanus glacialis is an essential food source for many economically important fish species in the Arctic.</td><td>Blachowiak-Samolyk et al., 2008; Søreide et al., 2008</td></tr>
<tr><td>Calanus finnmarchicus is less lipid rich than Calanus glacialis</td><td>Søreide et al., 2008</td></tr>
<tr><td>According to a model simulation of climate change scenarios in the Barents Sea, the Atlantic zooplankton species Calanus finnmarchicus increased approximately 20% and became more abundant in the east, while the Arctic zooplankton biomass (including Calanus glacialis) decreased 50%, causing the total simulated production to decrease.</td><td>Ellingsen et al. (2008)</td></tr>
<tr><td colspan="2">IEBM lens</td></tr>
<tr><td>Insights</td><td>There will potentially be a reduction in the quantity and quality of zooplankton available for fish production in the Barents Sea</td></tr>
<tr><td>Key research questions?</td><td>What are the implications of the reduction in quantity of zooplankton for fish production?

What are the implications of the reduction in quality of zooplankton for fish production?

What are the potential economic implications for the fisheries sector?

Does this change also impact on crabs, and if so how and how much?

Does the planktons vulnerability against pollution differ, and if so how?

How does this affect local livelihoods, indigenous peoples and the local fisheries industries?

Are there potential global repercussions?</td></tr>
</table>

Table S3: Scenario 2, increase in red king crab

Scenario 2. Increase in red king crab (*Paralithodes camtschaticus)* (biomass and expansion)		
**Background information**		**Reference**
The red king crab benefits from increased water temperatures in the Arctic		Stoner et al. 2010
The red king crab predates on capelin larvae		Mikkelsen 2013
Capelin is a key food species for other economically important fish species, e.g. cod		Gjøsæter et al. 2015
IEBM lens		
Insights	An increase in the biomass of red king crabs, due to increased water temperature, can potentially reduce capelin production and thus also impact on the production of other fish species e.g. cod.	
Key research questions?	What are the implications of a potential increase and spread of red king crab in the Arctic in the context of capelin production?<br>What are the implications of a potential decrease in capelin production on the production of other economically important fish species, e.g. cod?<br>What are the potential economic implications for the fisheries sector of cod and capelin?<br>What are the implications for the co-management strategies of the cod and capelin fisheries?<br>How does this affect local livelihoods, indigenous peoples and the local fisheries industries? Are those activities resilient to such change and could they seize the opportunity to produce King Crab instead?<br>Are there potential global repercussions?	

Table S4: Scenario 3, decrease in zooplankton

Scenario 3. Decrease in zooplankton production, (*Calanus glacialis*) due to mismatch	
Background information	Reference
The arctic grazer *Calanus glacialis* is an essential food source for many economically important fish species in the Arctic and indeed for the entire Arctic marine ecosystem. Among the zooplankton in the arctic shelf seas *Calanus glacialis* accounts for up to 80% of the biomass.	Blachowiak-Samolyk et al., 2008, Søreide et al. 2010
Ice algae is a key food source for *Calanus glacialis*, among many other species.	Søreide et al. 2010
There is a potential mismatch between the two primary production peaks of ice algae and the reproductive cycle of *Calanus glacialis*, due to the reduction in sea ice thickness and cover area driven by climate change.	Søreide et al. 2010

IEBM lens	
Insights	Due to the mismatch there could be a potential reduction in the biomass of *Calanus glacialis*, which in turn may affect fish production.
Key research questions?	What is the quantitative impact on zooplankton production of a potential mismatch?<br>What is the quantitative impact on fish production of a potential mismatch?<br>What are the potential economic implications for the fisheries sector?<br>Does this change also impact on crabs, and if so how and how much? Could crab fisheries replace traditional fisheries if there is a substantial drop in fish?<br>How does this affect local livelihoods, indigenous peoples and the local fisheries industries?<br>Are there potential global repercussions?

Table S5: Scenario 4, increase in European green crab

Scenario 4. Increase of the European green crab (C. maenas)	
**Background information**	**Reference**
The European green crab is one of the species potentially benefitting from increased shipping and transportation in the Arctic.	Roman and Palumbi (2004)
The green crab has been estimated to have the potential to expand to sub-Arctic and Arctic waters even under moderate climate change scenarios.	De Rivera et al. (2007)
The green crab needs a water temperature above 10°C to reproduce.	Cohen et al. 1995; Hines et al. 2004
The minimum water temperature for successful green crab reproduction is being approached in many places in the Arctic due to climate change.	Hines et al. 2004

IEBM lens	
Insights	Suitable areas for the European green crab are likely to expand in the Arctic due to increased shipping in combination with increased water temperatures, approaching minimum temperature for green crab reproduction. In many places European green crab is considered as a nuisance.
Key research questions?	What are the potential impacts of a spread of the green crab to new areas?<br>What are the implications for other crab species and for fish species?<br>What are the potential economic implications for the fisheries sector of cod and capelin?<br>How does this affect local livelihoods, indigenous peoples and the local fisheries industries? Are those activities resilient to such change?<br>Are there potential global repercussions?

Table S6: Scenario 5, increase in snow crab

Scenario 5 Continued increase in snow crab (*Chionoecetes bairdi*)		
**Background information**		**Reference**
There are ten times more snow crabs in the Barents Sea than red king crabs		Pettersen 2014
A large stock of Snow crabs could have a significant influence on the bottom communities where they forage – whether "good" or "bad" from the human point of view is difficult to predict.		Hjeltset 2014
Snow crabs does not seem to compete with fish for food and does not compete with the red king crab		Hjeltset 2014
The snow crab is food for cod		Hjeltset 2014
IEBM lens		
Insights	The snow crab has the potential to become an important economic species in the Arctic, but there are significant knowledge gaps on the impact of snow crabs on the Arctic ecosystem. Snow crab fisheries will potentially be of economic importance. Russia will start up a snow crab fishery 2014.	
Key research questions?	What impacts on the Arctic marine ecosystem can the snow crab have?<br>What impact on bottom communities can a high density of snow crabs have?<br>Can the snow crab become a significant food source for cod?<br>Can a market for snow crab fishery develop?<br>How does snow crab impact on local communities	

Table S7: Scenario 6: Ocean acidification

Scenario 6. Decrease in crab populations (red king crab and snow crab) due to ocean acidification	
**Background information**	**Reference**
Red king crab fisheries are economically important in the Arctic	Hjelstedt (2012)
Snow crab fisheries will potentially be of economic importance. Russia will start up a snow crab fishery 2014. There is now ten times as much snow crab than king crab in the Barents Sea	Pettersen 2014
The oceans have grown 30 percent more acidic since the beginning of the Industrial Revolution. Ocean acidification not only entails that the oceans become more acidic, but it also changes the sea's chemistry in other ways, such as robbing the water of important minerals that marine creatures need to grow, especially those with shells.	NOAA (2010)
The effects of long-term exposure to near-future levels of ocean acidification on the growth, condition, calcification, and survival of juvenile red king crabs (*Paralithodes camtschaticus*), and Tanner crabs ((snow crabs), (*Chionoecetes bairdi*))was examined and it was found that both species, survival decreased with pH, with 100% mortality of red king crabs occurring after 95 days in pH 7.5 water	Long et al. (2013)

IEBM lens	
Insights	There can be a potential reduction in crab production (snow crab and red king crab) due to increased ocean acidification.
Key research questions?	How large can the impact of ocean acidification on crab production be?<br>What can the economic implication of crab fisheries be?<br>What are the implications for future management strategies for fisheries in the Arctic?

References

ACCESS. 2012. Analysis and synthesis of extant and developing regulatory frameworks. Deliverable D5.11. http://www.access-eu.org/en/deliverables2/wp5.html.

ACCESS. 2013. 1st ACCESS summer school. Deliverable D6.251. https://wiki.met.no/_media/access/deliverables/d6-251-oasys.pdf

ACCESS. 2014a. Interactive noise maps of exploration / exploitation sites. Deliverable D4.51. http://www.access-eu.org/en/deliverables2/wp4.html

ACCESS. 2014b. Simulator of the effects of noise from oil industry operations on marine mammals. Deliverable D4.52.http://www.access-eu.org/en/deliverables2/wp4.html

ACCESS. 2014c. Infrastructure needs according to AMSA and other investigations. Deliverable D2.31. http://www.access-eu.org/en/deliverables2/wp2.html

ACCESS. 2015a. Operational conditions of an effective participation of Arctic indigenous people in the future Arctic governance. Deliverable D5.61. http://www.access-eu.org/en/deliverables2/wp5.html

ACCESS. 2015b. Summary of governance options over the ACCESS time period (ca 30 years) Deliverable D 5.41. http://www.access-eu.org/en/deliverables2/wp5.html

Blachowiak-Samolyk, K., J. E. Søreide, S. Kwasniewski, A. Sundfjord, H. Hop, S. Falk-Petersen, and E. Nøst Hegseth. 2008. Hydrodynamic control of mesozooplankton abundance and biomass in northern Svalbard waters (79-81°N). Deep-Sea Research Part II: Topical Studies in Oceanography 55:2210–2224.

Biggs, R., T. Blenckner, C. Folke, L. J. Gordon, A. Norström, M. Nyström, and G. D. Peterson. 2012. "Regime Shifts". In: Encyclopedia of Theoretical Ecology. Ed. by A. Hastings and L. Gross. Berkeley, United States: University of California Press.

Chapin, F. S. I., S. R. Carpenter, G. P. Kofinas, C. Folke, N. Abel, W. C. Clark, P. Olsson, D. M. S. Smith, B. H. Walker, O. R. Young, F. Berkes, R. Biggs, J. M. Grove, R. L. Naylor, E. Pinkerton, W. Steffen, and F. J. Swanson. 2010. Ecosystem stewardship: sustainability strategies for a rapidly changing planet. Trends in Ecology and Evolution 25:241–249.

Christensen, J.S, C.W. Mecklenburg and O.V. Karamushko, 2014. Arctic marine fishes and their fisheries in light of global change. *Global Change Biology.* 20(2): 352-359.

Cohen, A. N., J. T. Carlton, and M. C. Fountain. 1995. Introduction, dispersal and potential impacts of the green crab Carcinus maenas in San Francisco Bay, California. Marine Biology 122:225–237.

Corbett, J. J., D. A. Lack, J. J. Winebrake, S. Harder, J. A. Silberman, and M. Gold. 2010. Arctic shipping emissions inventories and future scenarios. Atmospheric Chemistry and Physics 10:9689–9704.

De Rivera, C.E., Steves, B.P., Ruiz, G.M., Fofonoff, P. & Hines, A. H. 2007. Northward spread of marine nonindigenous species along western North America: forecasting risk of colonization in Alaskan waters using environmental niche modeling. Page 36. Portland, United States.

Eide, A. 2017. Climate change, fisheries management and fishing aptitude affect spatial and temporal distributions in the Barents Sea cod fishery in Climate Change Economy and Society in the Arctic Ocean. Eds. Gascard, J.-C., A.-S. Crépin, M. Karcher and O.Young. eds. Special issue of *Ambio*.

Ellingsen, I. H., P. Dalpadado, D. Slagstad, and H. Loeng. 2008. Impact of climatic change on the biological production in the Barents Sea. Climatic Change 87:155–175.

Falk-Petersen, J. Paul Renaud and Natalia Anisimova. 2011. Establishment and ecosystem effects of the alien invasive red king crab (Paralithodes camtschaticus) in the Barents Sea – a review. CES Journal of Marine Science (2011), 68(3), 479–488. doi:10.1093/icesjms/fsq192.

Gjøsæter, H., Elvar H. Hallfredsson, Nina Mikkelsen, Bjarte Bogstad and Torstein Pedersen. 2015. Predation on early life stages is decisive for year-class strength in the Barents Sea capelin (*Mallotus villosus*) stock. ICES J. Mar. Sci. *(2015) doi: 10.1093/icesjms/fsv177*

J. M. Guinotte, V. J. Fabry. 2008. Ocean acidification and its potential effects on marine ecosystems. Ann. N. Y. Acad. Sci. 1134, 320–342. doi: 10.1196/annals.1439.013;pmid: 18566099

Hamilton, J. M., D. J. Maddison, and R. S. J. Tol. 2005a. Effects of climate change on international tourism. Climate Research 29:245–254.

Hamilton, J. M., D. J. Maddison, and R. S. J. Tol. 2005b. Climate change and international tourism: A simulation study. Global Environmental Change 15:253–266.

Hamre, J. 1994. Biodiversity and exploitation of the main fish stocks in the Norwegian - Barents Sea ecosystem.

Hjelset, A. M. 2014. Report from the workshop: Workshop on king- and snow crabs in the Barents Sea Tromsø, 11 – 12 March 2014.

Hjelstedt, A. 2012. Female life history parameters in the introduced red king crab (Paralithodes camtschaticus, Tilesius 1815) in the Barents Sea:A study of temporal and spatial variation in three Norwegian fjords. University of Tromsö.

Hines, Anson; Ruiz, Greg; Hitchcock, Natasha Gray; and de Rivera, Catherine E.,"Projecting Range Expansion of Invasive European Green Crabs (Carcinus maenas) to Alaska: Temperature and Salinity Tolerance of Larvae". 2004. Environmental Science and Management Faculty Publications and Presentations. Paper 77.http://pdxscholar.library.pdx.edu/esm_fac/77.

Hoegh-Guldberg, O., & Bruno, J. F. 2010. The impact of climate change on the world's marine ecosystems. *Science*, *328*(5985), 1523-1528.

Jørgensen, L.L. 2005. Biol Invasions. 7: 949. doi:10.1007/s10530-004-2996-1.

Jørgensen, L.L. & Primicerio, R. 2007. Hydrobiologia 590: 47. doi:10.1007/s10750-007-0756-9.

Kastner, T., M. J. I. Rivas, W. Koch, and S. Nonhebel. 2012. Global changes in diets and the consequences for land requirements for food.

Law, K., A. Roiger, J. Thomas, L. Marelle, J.-C. Raut, S. Dalsøren, J. Fuglestvedt, P. Tuccella, B. Weinzierl, and H. Schlager. 2017. Local Arctic air pollution: sources and impacts in Climate Change Economy and Society in the Arctic Ocean. Eds. Gascard, J.-C., A.-S. Crépin, M. Karcher and O.Young. eds. Special issue of *Ambio*.

Long, W. C., K. M. Swiney, C. Harris, H. N. Page, and R. J. Foy. 2013. Effects of Ocean Acidification on Juvenile Red King Crab (Paralithodes camtschaticus) and Tanner Crab (Chionoecetes bairdi) Growth, Condition, Calcification, and Survival. PLoS ONE 8.

Lutz, W., W. P. Butz, S. and Samir K.E. (eds.). 2014. World Population & Human Capital in the 21st Century. Oxford University Press, United Kingdom.

Mikkelsen, N. 2013. Predation on the demersal fish eggs of capelin Mallotus villosus and lumpsucker Cyclopterus lumpus in relation to recruitment. University of Tromsö.

Millennium Ecosystem Assessment (MA). 2005. Ecosystems and Human Well-being: Current State and Trends. Island Press, Washington DC, United States.

Morgenroth, E. 2014. Socio-economic costs and benefits of Arctic transport. ACCESS deliverable D2.61. http://www.access-eu.org/en/deliverables2/wp2.html.

NOAA. 2010. Ocean Acidification, Today and in the Future. https://www.climate.gov/news-features/featured-images/ocean-acidification-today-and-future [Accessed February, 2017]

Nordam, T. , D. A.E. Dunebier, C.J. Beegle-Krause, M. Reed and D. Slagstad. 2017. Impact of Climate Change and Seasonal Trends on the Fate of Arctic Oils spills in Climate Change Economy and Society in the Arctic Ocean. Eds. Gascard, J.-C., A.-S. Crépin, M. Karcher and O.Young. eds. Special issue of *Ambio*.

Olsen, E., Gjøsæter, H., Røttingen, I., Dommasnes, A., Fossum, P., & Sandberg, P. (2007). The Norwegian ecosystem-based management plan for the Barents Sea. *ICES Journal of Marine Science: Journal du Conseil*, *64*(4), 599-602.

Ospina, D. and A.S. Crépin. 2014. 2nd ACCESS Summer school. ACCESS deliverable D6.253 https://wiki.met.no/_media/access/deliverables/d6-253-beijer.pdf

Ottersen, G., K. Michalsen, and O. Nakken. 1998. Ambient temperature and distribution of north-east Arctic cod. Ices Journal of Marine Science 55:67–85.

Petrick, S., K. Riemann-Campe, S. Hoog, C. Growitsch, H. Schwind, R. Gerdes, and K. Rehdanz. 2017. Climate Change, Future Arctic Sea Ice and the Potential for European Arctic Offshore Oil and Gas Production in Climate Change Economy and Society in the Arctic Ocean. Eds. Gascard, J.-C., A.-S. Crépin, M. Karcher and O.Young. eds. Special issue of *Ambio*.

Pettersen, T. 2014, March 12. Snow crabs have found niche in Barents Sea ecosystem. http://barentsobserver.com/en/nature/2014/03/snow-crabs-have-found-niche-barents-sea-ecosystem-12-03.

Polyakov, I. V., J. E. Walsh, and R. Kwok. 2012. Recent changes of Arctic multiyear sea ice coverage and the likely causes. Bulletin of the American Meteorological Society 93:145–151.

Roman, J., and S. R. Palumbi. 2004. A global invader at home: Population structure of the green crab, Carcinus maenas, in Europe. Molecular Ecology 13:2891–2898.

Sætersdal, G., and H. Loeng. 1987. Ecological adaptation of reproduction in Northeast Arctic cod.

Sætre, R., R. Toresen, H. Søiland, and P. Fossum. 2002. The Norwegian spring-spawning herring - spawning, larval drift and larval retention. Sarsia: North Atlantic Marine Science 87:167–178.

Søreide, J. E., S. Falk-Petersen, E. N. Hegseth, H. Hop, M. L. Carroll, K. A. Hobson, and K. Blachowiak-Samolyk. 2008. Seasonal feeding strategies of Calanus in the high-Arctic Svalbard region. Deep-Sea Research Part II: Topical Studies in Oceanography 55:2225–2244.

Søreide, J. E., E. V. A. Leu, J. Berge, M. Graeve, and S. Falk-Petersen. 2010. Timing of blooms, algal food quality and Calanus glacialis reproduction and growth in a changing Arctic. Global Change Biology 16:3154–3163.

Stammler-Gossmann, A. 2014. Economic settings, societal and cultural priorities in the fishery and aquaculture sectors past and present impact of biophysical changes on fisheries. ACCESS deliverable D3.41. http://www.access-eu.org/en/deliverables2/wp3.html

Steffen, W., P. J. Crutzen, and J. R. McNeill. 2007. The Anthropocene: Are Humans Now Overwhelming the Great Forces of Nature. AMBIO: A Journal of the Human Environment 36:614–621.

Stephens, D. W., and J. R. Krebs. 1986. Foraging theory. Princeton University Press, Princeton, NJ.

Stoner, A.W., M.L. Ottmar, and L.A. Copeman. 2010. Temperature effects on the molting, growth, and lipid composition of newly-settled red king crab. *Journal of Experimental Marine Biology and Ecology*, **393**, 138–147.

Stroeve, J. C., V. Kattsov, A. Barrett, M. Serreze, T. Pavlova, M. Holland, and W. N. Meier. 2012a. Trends in Arctic sea ice extent from CMIP5, CMIP3 and observations. Geophysical Research Letters 39.

Stroeve, J. C., M. C. Serreze, M. M. Holland, J. E. Kay, J. Malanik, and A. P. Barrett. 2012b. The Arctic's rapidly shrinking sea ice cover: A research synthesis. Climatic Change 110:1005–1027.

Troell, M, A. Eide, J. Isaksen, Ø. Hermansen, and A.-S Crépin. 2017. Seafood from a changing Arctic in Climate Change Economy and Society in the Arctic Ocean. Eds. Gascard, J.-C., A.-S. Crépin, M. Karcher and O.Young. eds. Special issue of *Ambio*.

Toresen, R., and O. J. Østvedt. 2000. Variation in abundance of Norwegian spring-spawning herring (Clupea harengus, Clupeidae) throughout the 20th century and the influence of climatic fluctuations. Fish and Fisheries 1:231–256.

Vedenev, A., M. van der Schaar, and M. André. 2014. Noise propagation from commercial fishing and vessel traffic in the Arctic today and in the future. ACCESS deliverable D2.44. http://www.access-eu.org/en/deliverables2/wp2.html

Wadhams, P. 2012. Arctic ice cover, ice thickness and tipping points.

Wadhams, P., N. Hughes, and J. Rodrigues. 2011. Arctic sea ice thickness characteristics in winter 2004 and 2007 from submarine sonar transects. Journal of Geophysical Research: Oceans 116.

Wang, M., and J. E. Overland. 2012. A sea ice free summer Arctic within 30 years: An update from CMIP5 models. Geophysical Research Letters 39.

Ware, C., J. Berge, J. H. Sundet, J. B. Kirkpatrick, A. D. M. Coutts, A. Jelmert, S. M. Olsen, O. Floerl, M. S. Wisz, and I. G. Alsos. 2014. Climate change, non-indigenous species and shipping: Assessing the risk of species introduction to a high-Arctic archipelago. Diversity and Distributions 20:10–19.

Wilkinson, J., C.J. Beegle-Krause, K.-U. Evers, N. Hughes, A. Lewis, M. Reed, and P. Wadhams. 2017. Oil spill response capabilities and technologies for ice-covered Arctic marine waters: A review of recent developments and established practices.

Ødemark, K., S. B. Dalsøren, B. H. Samset, T. K. Berntsen, J. S. Fuglestvedt, and G. Myhre. 2012. Short-lived climate forcers from current shipping and petroleum activities in the Arctic. Atmospheric Chemistry and Physics 12:1979–1993.
